# Supplementary material for: Synthesis of Eco-Friendly Biopolymer, Alginate-Chitosan Composite to Adsorb the Heavy Metals, Cd(II) and Pb(II) from Contaminated Effluents
Source: Materials (Basel). 2021 Apr 24;14(9):2189. doi: 10.3390/ma14092189 (PMC8123203; doi:10.3390/ma14092189)
Supplement: Supplementary file 1 [file materials-14-02189-s001.zip › materials-1191256-supplementary.pdf]

materials

MDPI

Supplementary Materials

Synthesis of Eco-Friendly Biopolymer, Alginate-Chitosan Composite to Adsorb the Heavy Metals, Cd(II) and Pb(II) from Contaminated Effluents

Mohammed F. Hamza [1,2,*], Nora A. Hamad [3], Doaa M. Hamad [3], Mahmoud S. Khalafalla [2], Adel A.-H. Abdel-Rahman [3], Ibrahim F. Zeid [3], Yuezhou Wei [1,4,*], Mahmoud M. Hessien [5], Amr Fouda [6] and Waheed M. Salem [7]

[1] Guangxi Key Laboratory of Processing for Non-ferrous Metals and Featured Data, School of Resources, Environment and Data, Guangxi University, Nanning 530004, China
[2] Nuclear Materials Authority, P.O. Box. 530, El-Maadi, Cairo 11381, Egypt; mahmoudsayed24@yahoo.com
[3] Department of Organic Chemistry, Faculty of Science, Menoufia University, Shebine El-Koam 00123, Egypt; nhamad059@gmail.com (N.A.H.); dodyhamad95@gmail.com (D.M.H.); adelnassar63@yahoo.com (A.A.-H.A.-R.); ibrahimzeid296@gmail.com (I.F.Z.)
[4] School of Nuclear Science and Engineering, Shanghai Jiao Tong University, Shanghai 200240, China
[5] Department of Chemistry, College of Science, Taif University, P.O. Box. 11099, Taif 21974, Saudi Arabia; m.hessien@tu.edu.sa
[6] Botany and Microbiology Department, Faculty of Science, AL-Azhar University, Cairo 11884, Egypt; amr_fh83@azhar.edu.eg
[7] Medical Laboratories Department, Faculty of Applied Health Science technology, Menoufia University, Shebine El-Koam 32511, Egypt; waheedsalem1979@gmail.com
* Correspondence: m_fouda21@hotmail.com (M.F.H.); yzwei@gxu.edu.cn (Y.W.)

Table S1. Maximum contaminant limits (MCL) and Recommended Maximum Concentrations (RMC) of some heavy metals in drinking water, irrigation water, and livestock drinking water.

Element	MCLs (mg L^{-1})			RMC (mg L^{-1})	
	EU [1]	USEPA [2]	WHO [3]	Irrigation Water	Livestock Drinking Water
Cadmium	0.005	0.005	0.003	0.01	0.05
Lead	0.1	0.015	0.005	5.0	0.1
Copper	–	1.3	2.0	0.20	0.5
Zinc	–	5.0	5.0	2.0	24.0
Nickel	–	–	0.07	0.20	–
Aluminum	–	0.2	–	5.0	5.0
Iron	–	0.3	–	5.0	–

Table S2. Uptake kinetics modeling—PFORE (pseudo-first-order rate equation), PSORE (pseudo-second-order rate equation), and RIDE (resistance to intraparticle diffusion equation—Crank equation).

Model	Equation	Parameters	
PFORE [4]	$q(t) = q_{eq,1}(1 - e^{k_1 t})$	$q_{eq,1}$ (mg g^{-1})	k_1 (min^{-1})
PSORE [4]	$q(t) = \frac{q_{eq,2}^2 \times k_2 \times t}{1 + q_{eq,2} \times k_2 \times t}$	$q_{eq,2}$ (mg g^{-1})	k_2 (L mg^{-1} min^{-1})

Table S3. Sorption isotherm modeling [5,6].

Model	Langmuir	Freundlich	Sips
Equation	$q = \frac{q_{m,L} \times b_L \times C_{eq}}{1 + b_L \times C_{eq}}$	$q = k_F\, C_{eq}^{1/n}$	$q = \frac{q_{m,S} \times b_S \times {C_{eq}}_{eq}^{1/n_S}}{1 + b_S \times {C_{eq}}_{eq}^{1/n_S}}$
Parameters	$q_{m,L}$ (mg g^{-1})*	k_F (mg$^{1-1/n}$ g^{-1} L$^{-1/n}$)	$q_{m,S}$ (mmol g^{-1})*
	b_L (L mg^{-1})**	n (dimensionless)	b_S (L mg^{-1})**
	–	–	n_S (dimensionless)

* Sorption capacity at saturation of the monolayer; ** Affinity coefficient

Akaike Information Criterion, AIC [7]:

$$AIC = N \ln\left(\frac{\sum_{i=0}^{N}\left(y_{i,exp.} - y_{i,model}\right)^2}{N}\right) + 2N_p + \frac{2N_p\left(N_p + 1\right)}{N - N_p - 1} \quad (1)$$

where N is the number of experimental points, N_P is the number of model parameters, $y_{i,exp.}$ and $y_{i,\,model}$ are the experimental and calculated values of the tested variable

Table S4. Maximum contaminant limits (MCL) of some heavy metals in drinking water.

Metal	Co	MCL* (mg L^{-1})	C_0/MCL ratio
Cu	6.403	2	3.2015
Hg	0.83	0.002	415
Fe	165.4	0.3	551.33333
Al	125.8	0.2	629
Zn	7.39	5	1.478
Cd	1.96	0.003	653.33333
Pb	2.03	0.01	203
Ni	2.18	0.07	31.142857

*Maximum contaminant limits (MCL) according to WHO, while Al and Fe are assigned according to the US.EPA

Table S5a. XPS analysis (HRES XPS peaks) of C 1s, N 1s, O 1s, Cl 2p, M (M = Cd and Pb) for CA#3 sorbent before and after metal sorption.

Element	Raw composite (CA#3)	CA#3 + Pb	CA#3 + Cd
C 1s	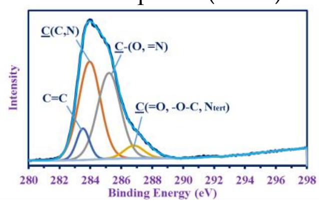	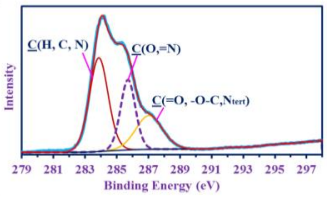	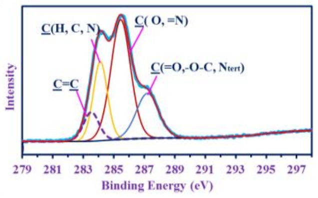
O 1s	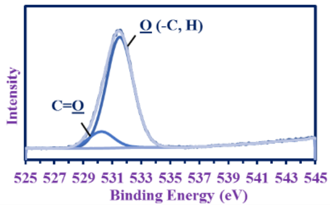	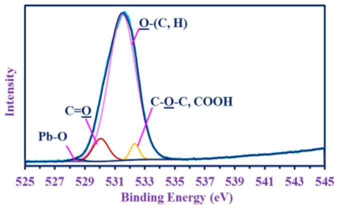	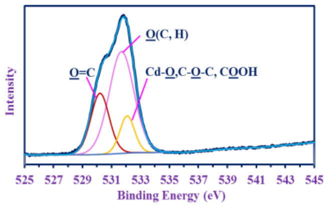

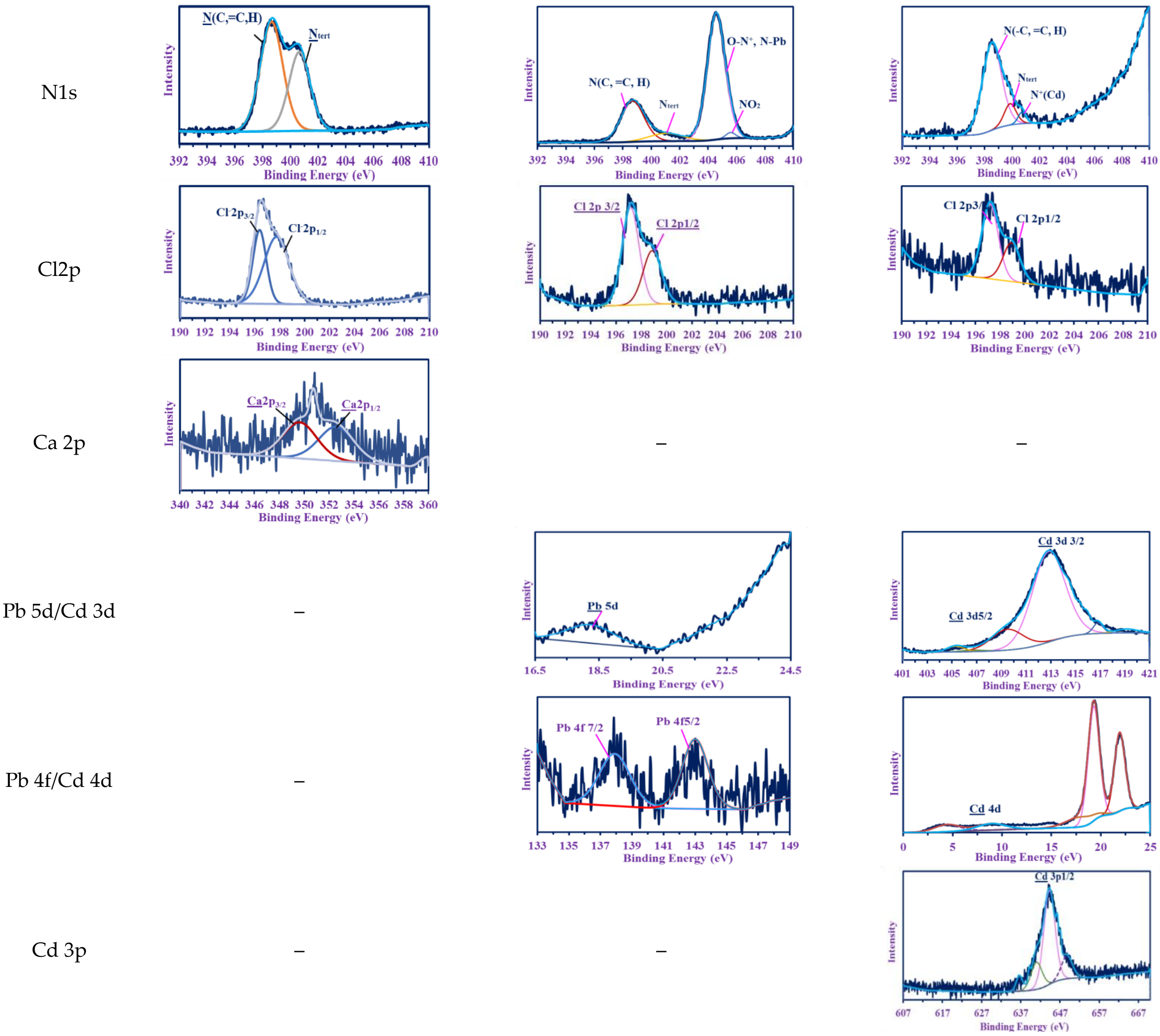

Table S5b. Assignment peaks, BEs, FWHE, and atomic fractions (AF, %) of C 1s, N 1s, O 1s, Cl 2p, M (M=Cd and Pb) for CA#3 sorbent before and after metal sorption.

Element/ Transition peak				Pb			Cd			
	BE	FWHE	AF%	BE	FWHE	AF%	BE	FWHE	AF%	
C 1s	284.35	1.67	44.56	284.17	0.86	31.14	284.12	1.1	22.81	C(C,H,N)
	285.82	1.76	40.49	285.14	0.84	30.96	285.46	1.52	47.76	C(O,=N)
	286.81	1.52	8.06	287.05	1.98	37.9	287.21	1.72	20.28	C(=O,-O-,Ntert)
	283.93	1.91	6.89	–	–	–	283.53	1.2	9.14	C=C
O 1s	530.24	2.05	12.47	530.05	1.23	7.92	530.21	1.5	26.83	C=O
	531.51	2.16	87.53	531.5	2.13	88.12	531.69	2.02	60.14	O(C,H)
	–	–	–	532.32	0.7	3.22	532.09	1.2	13.03	CO(C,OH)/O-Cd
				528.47	1.09	0.73	–	–	–	Pb-O
N 1s	398.64	1.92	59.63	398.68	1.9	26.61	398.51	1.64	82.87	N(C,=C,H)
	400.66	1.8	40.37	401.06	2.81	7.58	399.85	1.03	12.67	N_{tert}
	–	–	–	404.57	1.47	64.2	400.71	0.67	4.46	$O-N^+$,N(M)
				405.59	0.8	1.61	–	–	–	NO_2

Ca 2p	349.64	3.25	50.83	–	–	–	–	–	–	Ca 2p 3/2
	352.6	3.35	49.17	–	–	–	–	–	–	Ca 2p 1/2
Cl 2p	196.59	1.26	36.66	197.05	1.57	62.41	197.07	1.57	64.84	Cl 2p3/2
	197.75	2.39	63.34	198.56	1.73	37.59	198.63	1.66	35.16	Cl 2p 1/2
Cd 3d		–		–	–	–	405.29	1.65	4.38	Cd 3d5/2
							409.48	3.09	20.77	
							412.84	3.12	69.89	Cd 3d1/2
							416.83	0.94	4.96	
Cd 3p		–		–	–	–	636.61	1.86	5.39	Cd 3p1/2
							641.01	3.33	18.97	
							644.28	3.36	59.03	
							648.74	3.33	16.61	
Cd 4d		–		–	–	–	4.43	3.08	6.63	Cd 4d
							8.95	3.36	6.65	
							17.89	3.36	10.11	–
							19.29	1.34	46.34	–
							21.91	1.27	30.26	–
Pb 5d		–		18.23	1.73	100	–	–	–	Pb 5d
Pb 4f		–		143	1.92	52.35	–	–	–	Pb 4f5/2
		–		138.1	2.31	47.65	–	–	–	Pb 4f7/2

Table S6. EA of CA#2, CA#3, and CA#4 sorbents for C, N, O, and H%.

**Sorbent**	**C**	**H**	**O**	**O (mmol)**	**N**	**N (mmol)**
CA#2	32.94	4.74	29.82	18.64	6.33	4.52
CA#3	35.9	4.49	33.58	20.99	5.22	3.73
CA#4	37.87	4.32	38.09	23.80	4.19	2.99

Table S7. Ionic properties of selected metals.

**Parameter**	**Pb(II)**	**Cd(II)**
Ionic radius (Å) of hydrated species	1.20 Å [8]	0.95-0.96 Å [9]
Diffusivity in water ($\times 10^5$ cm^2 s^{-1})	0.939 [10]	0.719 [11]
Softness	+0.41 [11]	+0.58 [11]

Table S8. Selectivity Coefficient (SC), K_d values, C_o, C_f and q_{eq} of Cd and Pb in equi-molar solution containing Al, Ca, and Mg as a function of pH.

Elements	**pHeq**	**C_o mmol L^{-1}**	**C_f mmol g^{-1}**	**q_{eq} mmol g^{-1}**	**K_d**	**Selectivity Coefficient**	
						**Cd/M**	**Pb/M**
Al	2.43	1.105	1.038	0.079	0.077	0.969	0.681
	3.68	1.105	1.037	0.08	0.078	5.552	3.148
	4.22	1.105	1.028	0.091	0.089	25.8	8.198
	4.76	1.105	1.014	0.108	0.106	22.65	6.31
Ca	2.43	1.042	0.998	0.052	0.053	1.411	0.991
	3.68	1.042	0.99	0.063	0.063	6.7912	3.851
	4.22	1.042	0.951	0.109	0.114	19.948	6.339
	4.76	1.042	0.937	0.126	0.135	17.892	4.983
Mg	2.43	1.061	1.035	0.031	0.03	2.496	1.753
	3.68	1.061	1.003	0.069	0.069	6.200	3.516
	4.22	1.061	0.965	0.115	0.119	19.229	6.109
	4.76	1.061	0.921	0.1673	0.182	13.28	3.699
Cd	2.43	1.098	1.034	0.0767	0.074	–	0.702
	3.68	1.098	0.807	0.348	0.431	–	0.567
	4.22	1.098	0.377	0.862	2.287	–	0.318
	4.76	1.098	0.364	0.878	2.412	–	0.279
	2.43	1.051	1.005	0.052	0.052	1.424	–

Pb	3.68	1.051	0.873	0.213	0.244	1.764	–
	4.22	1.051	0.653	0.475	0.727	3.147	–
	4.76	1.051	0.673	0.452	0.672	3.59	–

Table S9. Comparison of the metal concentration with the MCL (mg L^{-1}) according to drinking water guides and the removal efficiency at different pH values.

pH	Metal	Co (mg L^{-1})	Ceq (mg L^{-1})	MCL (mg L^{-1})	Removal Eff. (%)	C_0/MCL Ratio	C_{eq}/MCL Ratio
2	Cu	6.403	5.12	2	20.04	3.20	2.56
	Hg	0.83	0.384	0.002	53.74	415	192
	Fe	165.4	73	0.3	55.87	551.33	243.33
	Al	125.8	54.8	0.2	56.44	629	274
	Zn	7.39	6.91	5	6.49	1.48	1.38
	Cd	1.96	1.81	0.003	7.65	653.33	603.33
	Pb	2.03	1.83	0.01	9.85	203	183
	Ni	2.18	2.081	0.07	4.54	31.14	29.73
4.04	Cu	6.403	1.98	2	69.08	3.20	0.99
	Hg	0.83	0.194	0.002	76.63	415	97
	Fe	165.4	10.95	0.3	93.38	551.33	36.5
	Al	125.8	35.9	0.2	71.46	629	179.5
	Zn	7.39	3.35	5	54.67	1.48	0.67
	Cd	1.96	0.26	0.003	86.73	653.33	86.67
	Pb	2.03	0.56	0.01	72.41	203	56
	Ni	2.18	1.07	0.07	50.92	31.14	15.29
5.8	Cu	6.403	0.93	2	85.48	3.20	0.46
	Hg	0.83	0.014	0.002	98.31	415	7
	Fe	165.4	5.05	0.3	96.95	551.33	16.83
	Al	125.8	11.15	0.2	91.14	629	55.75
	Zn	7.39	1.31	5	82.27	1.478	0.26
	Cd	1.96	0.011	0.003	99.44	653.33	3.67
	Pb	2.03	0.016	0.01	99.21	203	1.6
	Ni	2.18	0.095	0.07	95.64	31.14	1.36

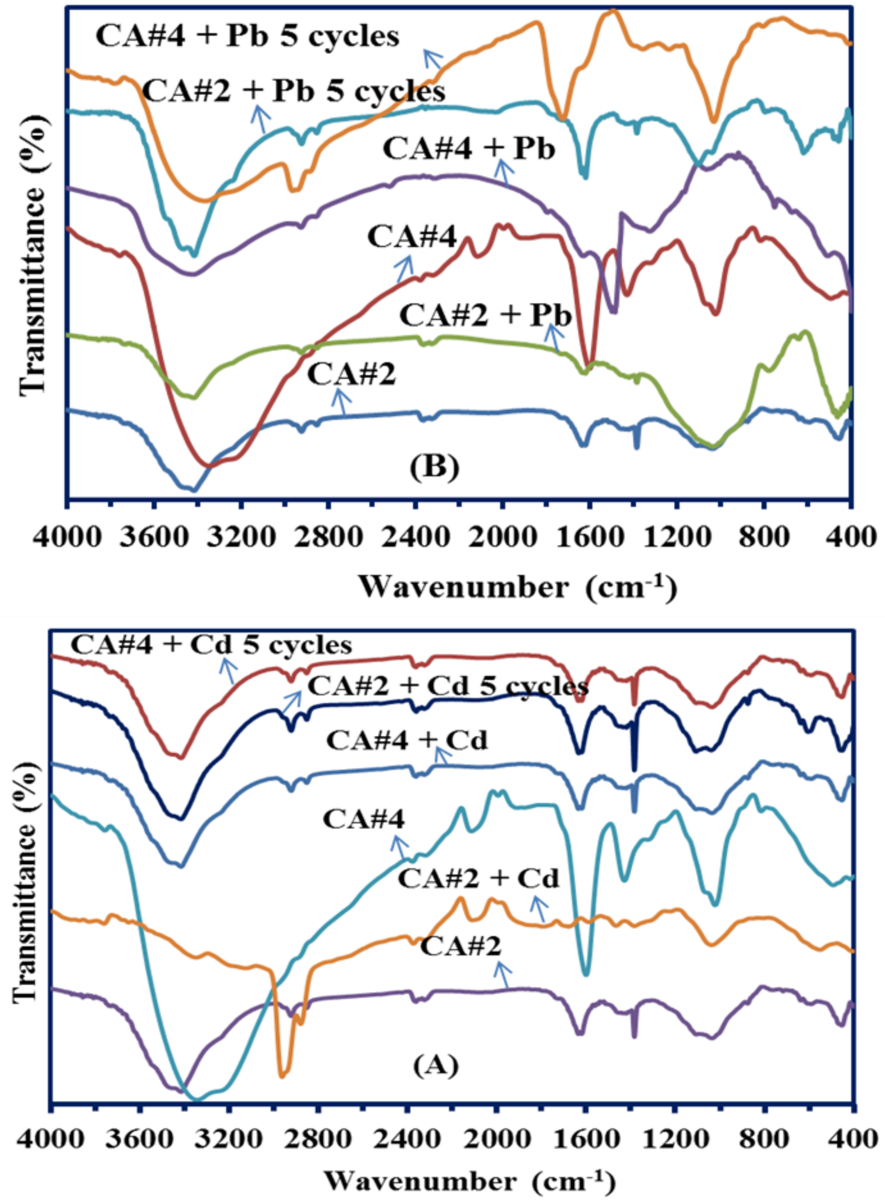

Figure S1. FTIR analyses of CA#2 and CA#4 of loaded metal ions (Cd (A) and Pb (B)), after desorption, and after 5 cycles of sorption desorption.

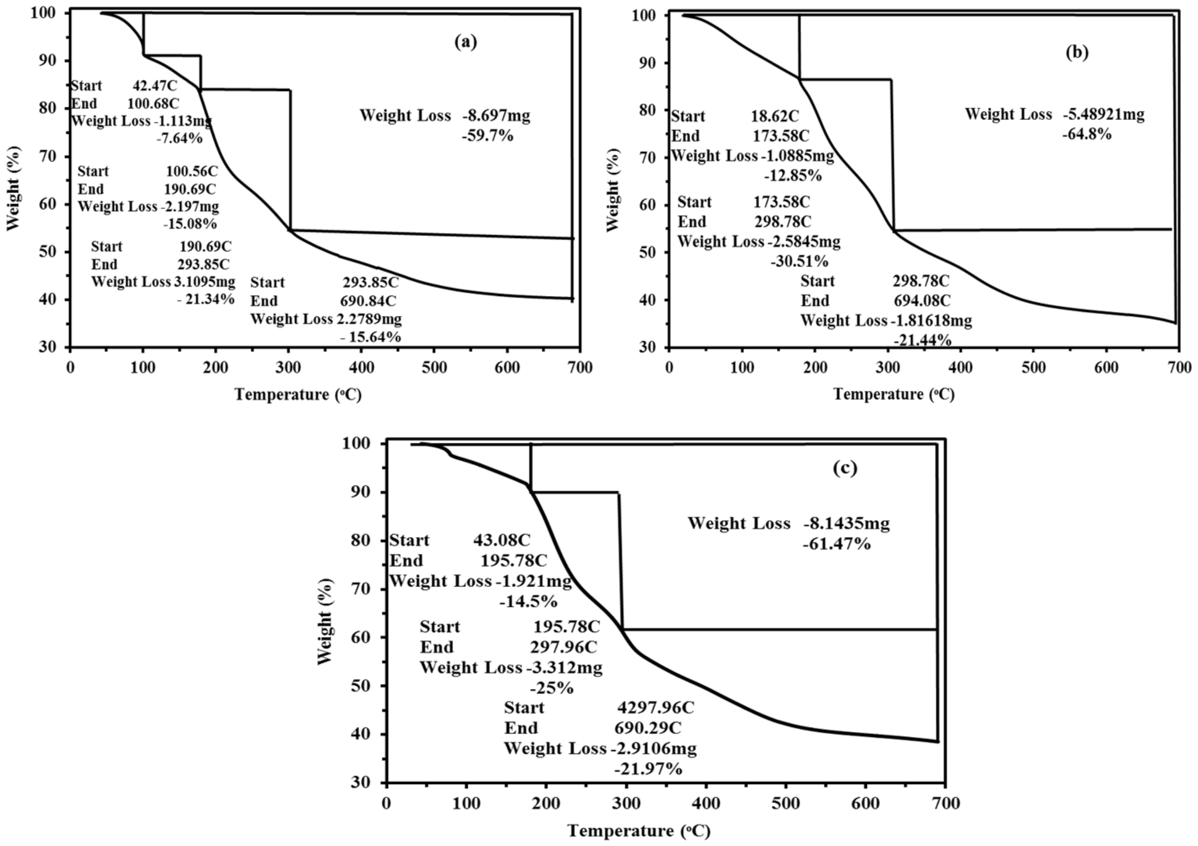

Figure S2a. TGA analysis of CA#2-(**a**), CA#3-(**b**), and CA#4-(**c**) sorbents.

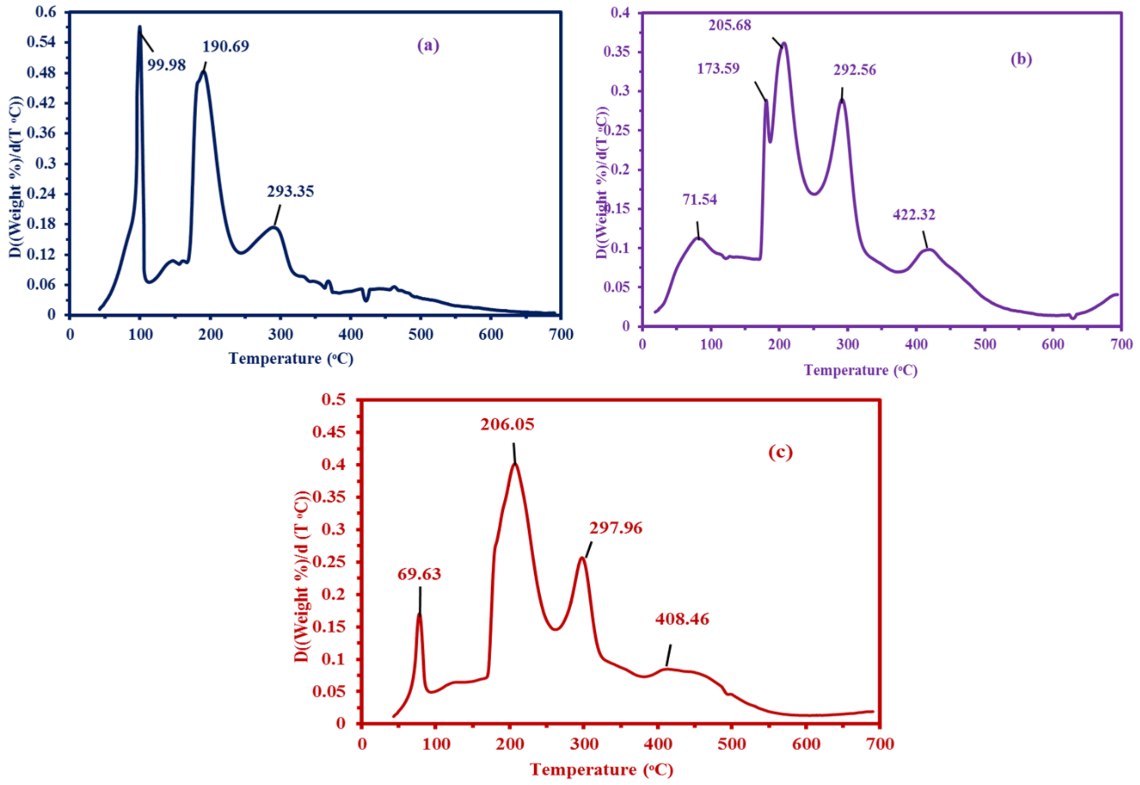

Figure S2b. DTG of the synthesized CA#2-(**a**), CA#3-(**b**), and CA#4-(**c**) sorbents.

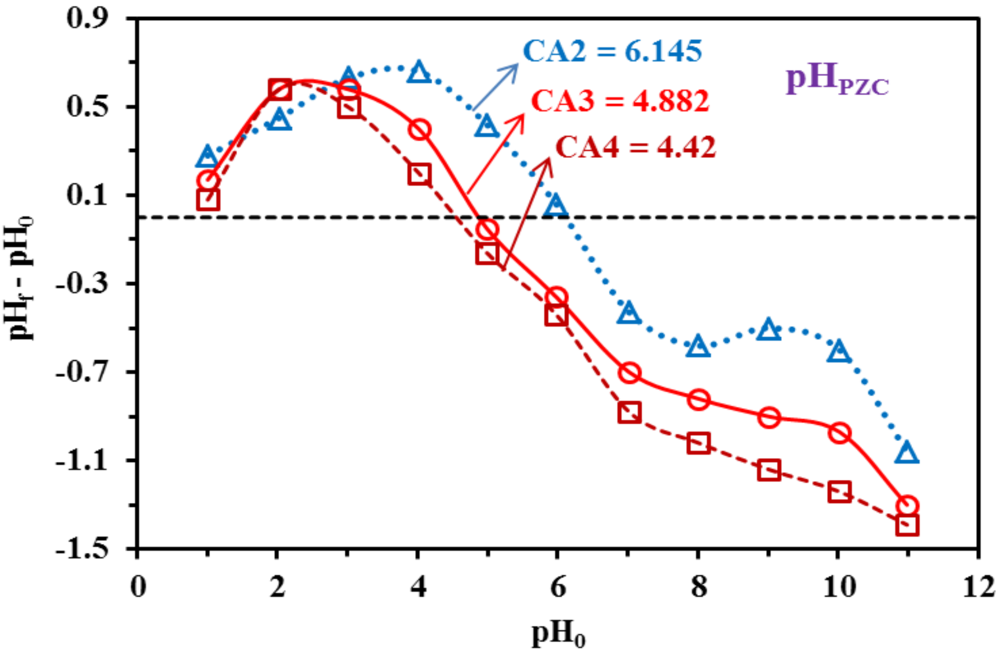

Figure S3. pH_{pzc} measurements of CA#2, CA#3, and CA#4 sorbents (SD: 2 g L^{-1}; time of agitation: 48 h; speed, 170 (±3) rpm; T: 23 (±3) °C).

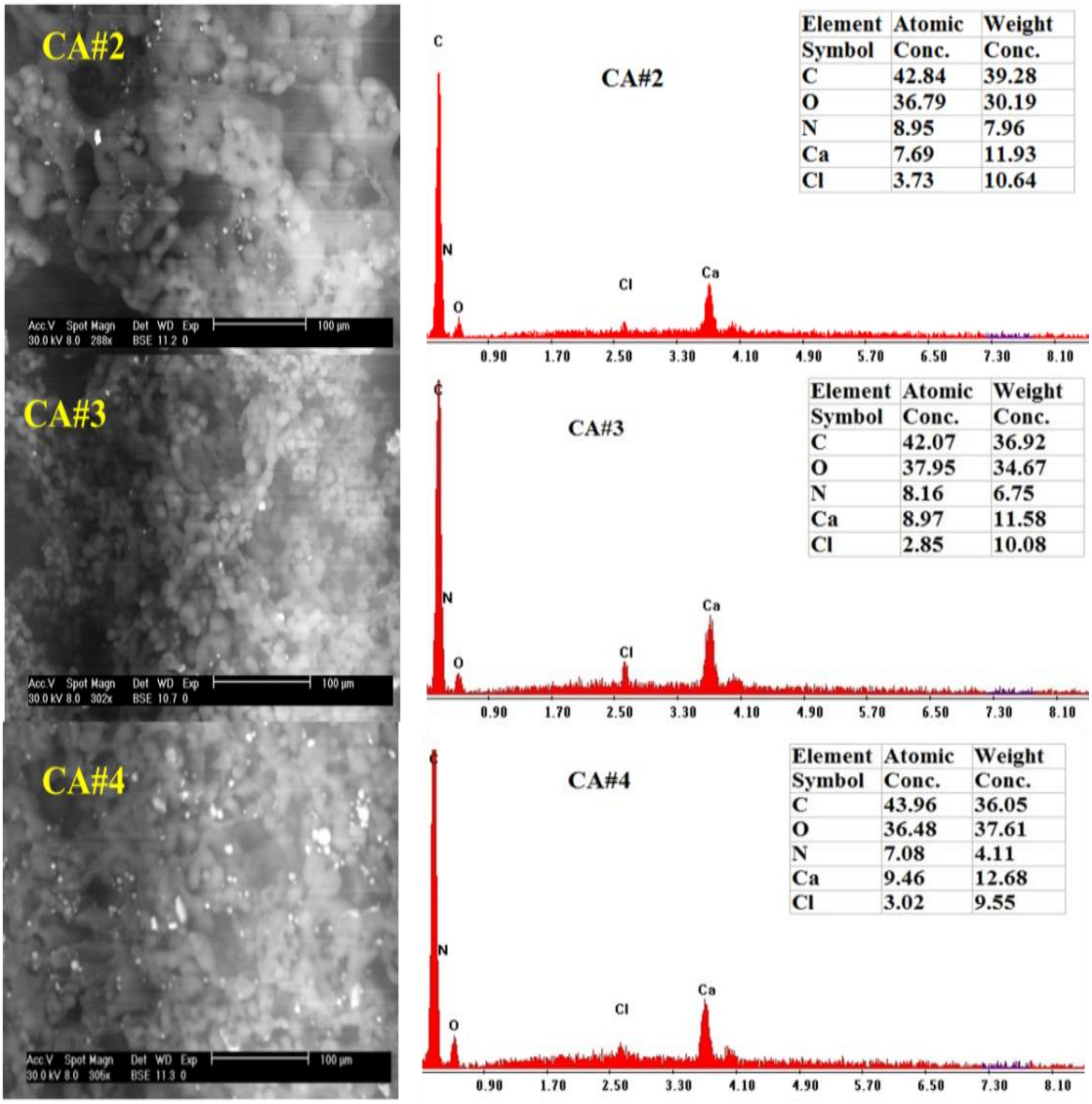

Figure S4. SEM and EDX for the semi-quantitative analysis of CA#2, CA#3, and CA#4 sorbents.

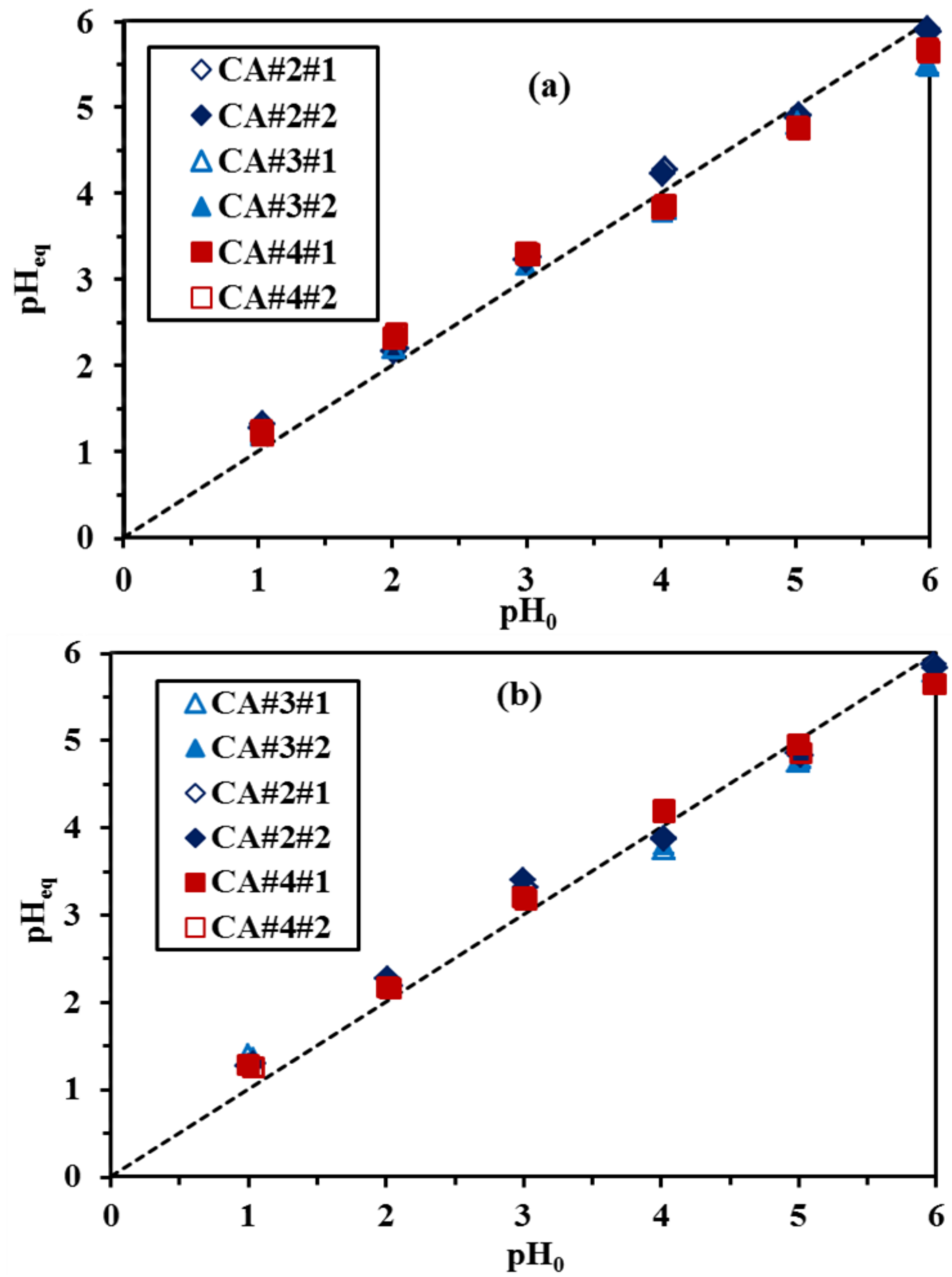

Figure 5. pH variation for Cd(II)-(a) and Pb(II)-(b) sorption using CA#2, CA#3, and CA#4 sorbents (C_0: 0.958 mmol Cd L^{-1} for 1st Series and 0.967 mmol Cd L^{-1}—2nd Series, C_0: 0.48 mmol Pb L^{-1} for—1st Series and 0.51 mmol Pb L^{-1}—2nd Series, sorbent dosage, SD: 1 g L^{-1}; temperature, T: 23 (±3) °C; time: 48 h; agitation speed: 170 (±3) rpm).

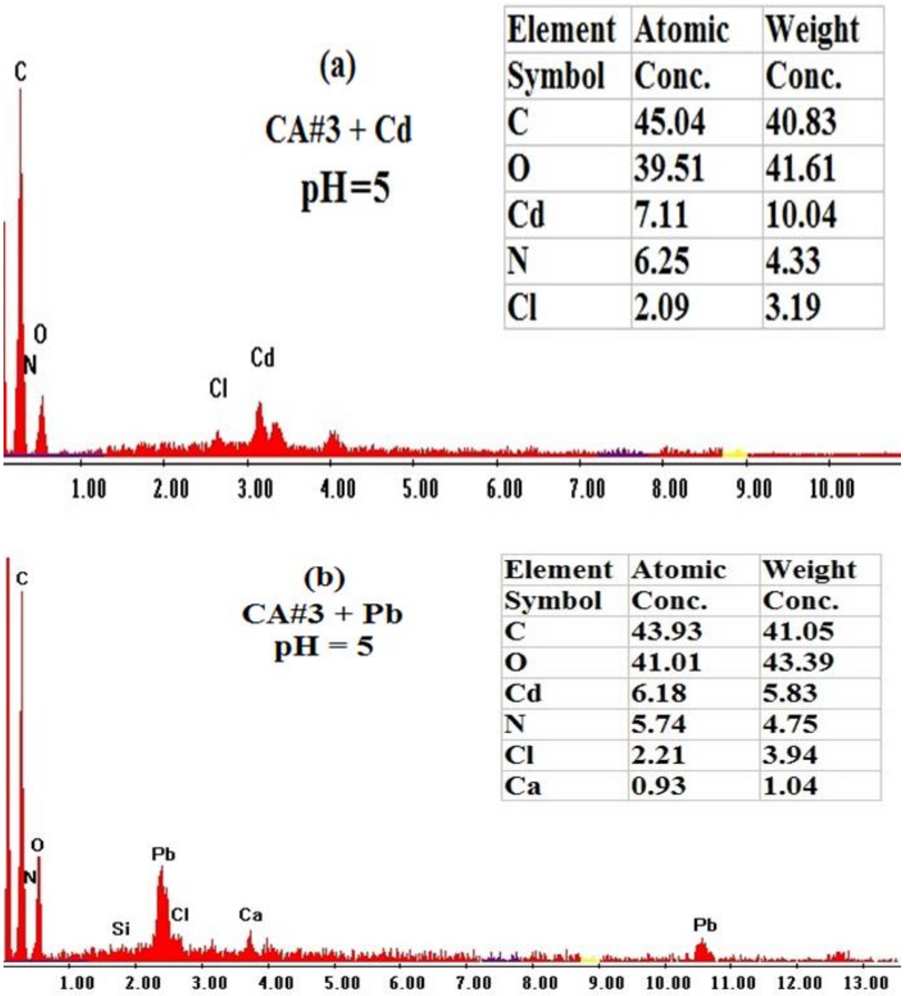

Element Symbol	Atomic Conc.	Weight Conc.
C	45.04	40.83
O	39.51	41.61
Cd	7.11	10.04
N	6.25	4.33
Cl	2.09	3.19

Element Symbol	Atomic Conc.	Weight Conc.
C	43.93	41.05
O	41.01	43.39
Cd	6.18	5.83
N	5.74	4.75
Cl	2.21	3.94
Ca	0.93	1.04

Figure S6. SEM-EDX analysis of sorbent (CA#3) after loading with Cd(II) -(**a**) and Pb(II) -(**b**) ions.

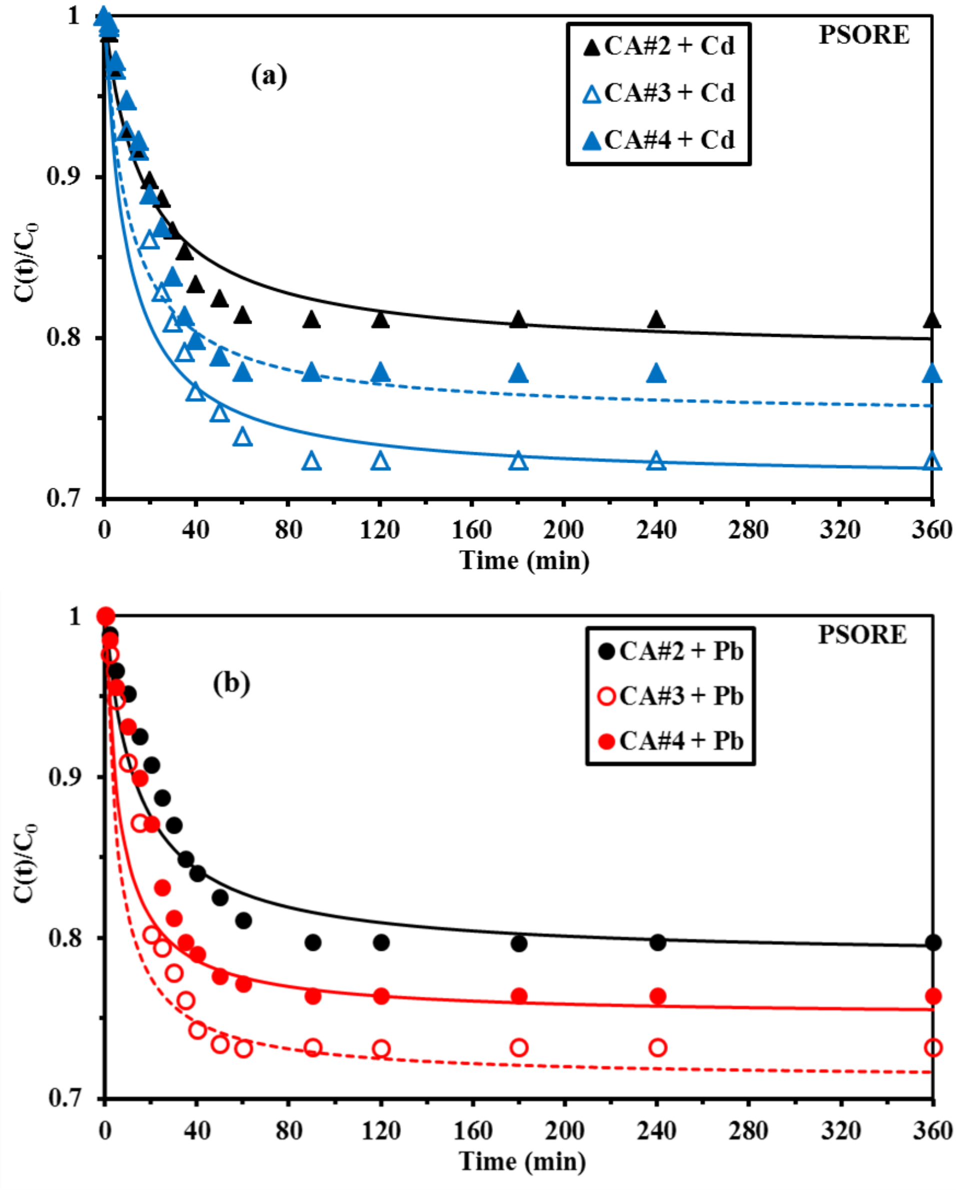

Figure S7. Sorption kinetics profile of the PSORE for Cd(II) (**a**) and Pb(II) (**b**) (C_0: 100 (±5) mg L^{-1}; SD: 250 mg L^{-1}, pH_0: 5).

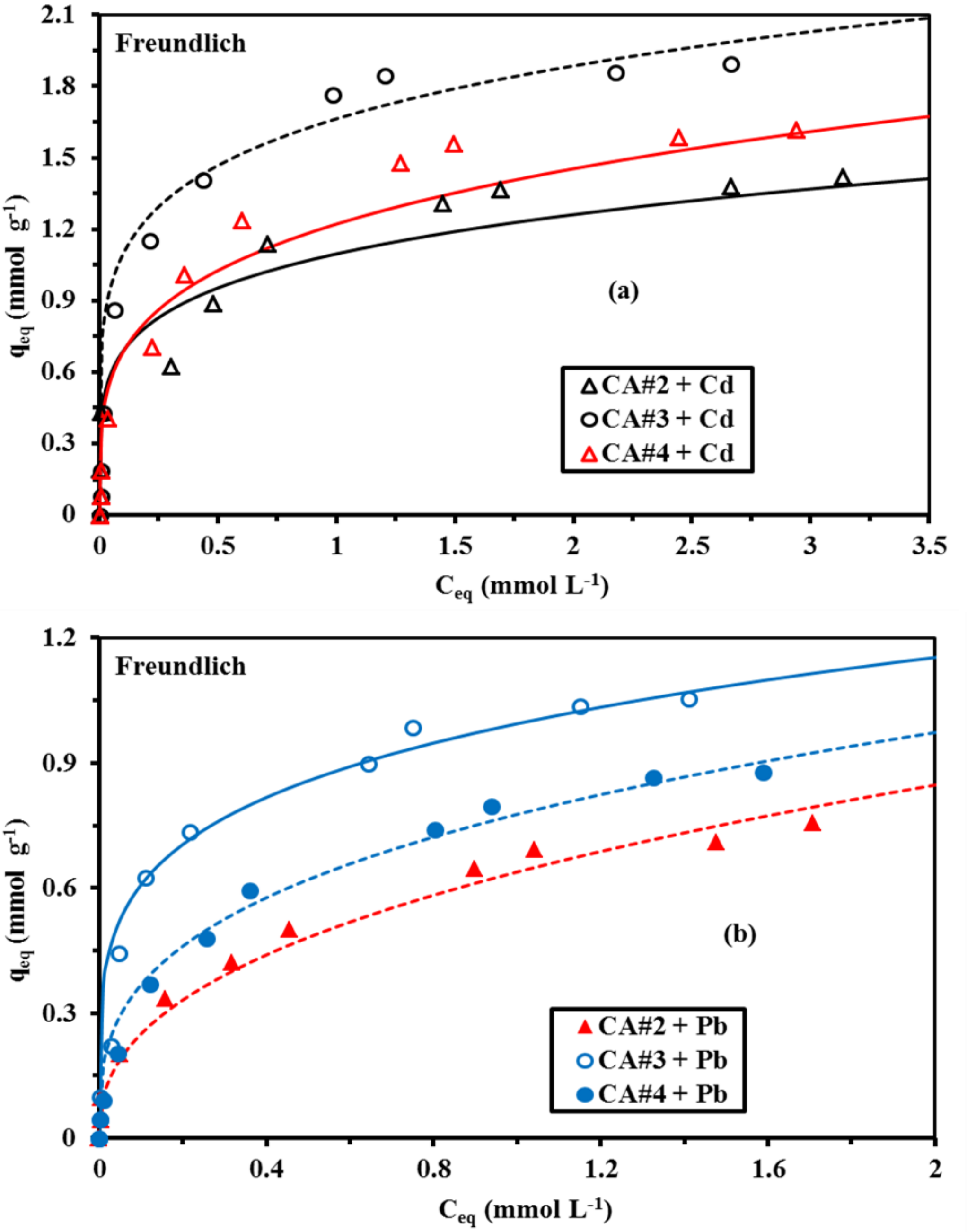

Figure S8. Cd (II), (**a**) and Pb(II), (**b**) sorption isotherms using CA#2, CA#3, and CA#4—Modeling with Freundlich equation (C_0: 10–500 mg M L^{-1}; SD: 1 g L^{-1}; T: 23 (±3) °C; time: 48 h; agitation speed: 170 (±3) rpm).

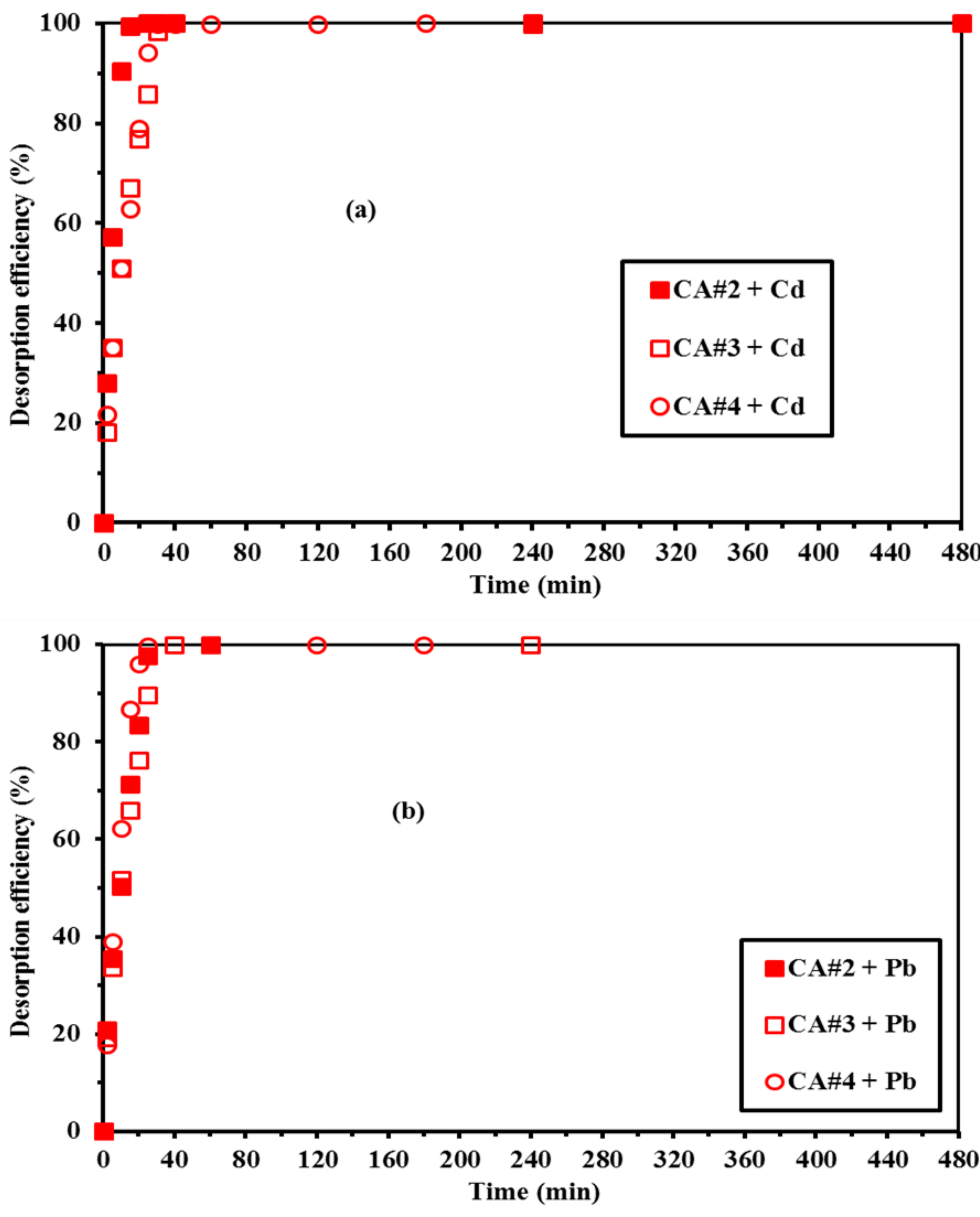

Figure S9. Desorption efficiencies for loaded sorbent (from kinetic experiments) for Cd(II) (a) and Pb(II) (b)-using 0.2M HCl (SD: 1 g L^{-1}; T: 23 (±3) °C; time: 24 h; agitation speed: 170 (±3) rpm).

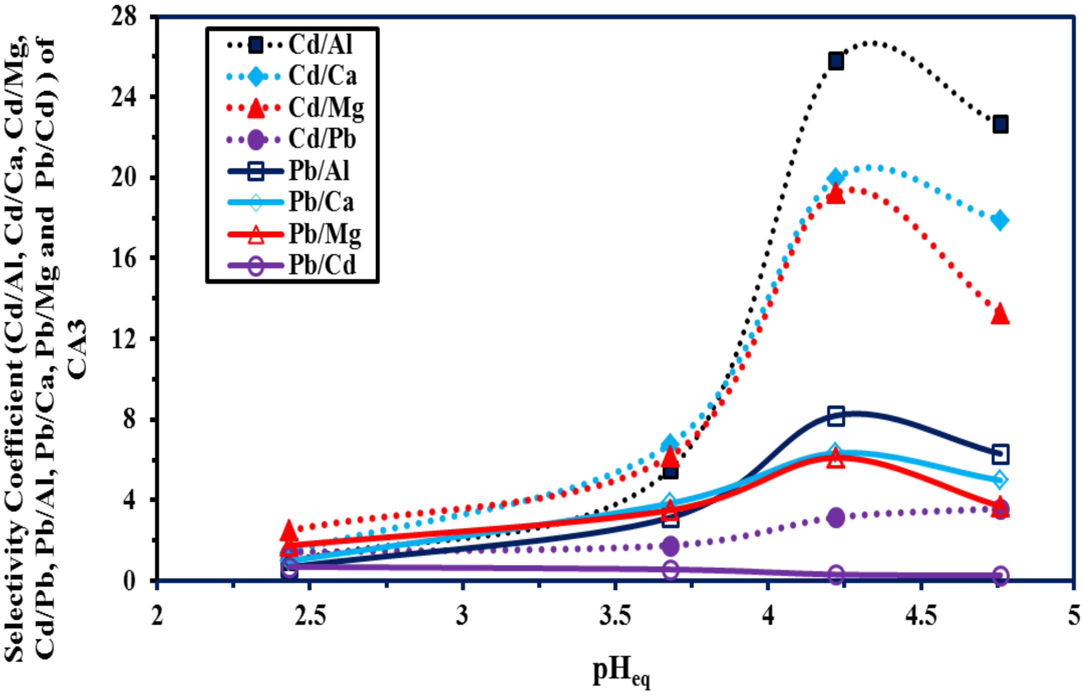

Figure S10. Sorption of Cd (solid symbols/dotted lines) and Pb (open symbols/solid lines) comparing to other elements in multi-component of mono, di and trivalent cations (multi-component concentrations; C_0 (M): 1.0 mmol metal L^{-1}; SD: 1 g L^{-1}; time of agitation: 48 h; speed: 170 (±3) rpm; T: 23 ± 3 °C).

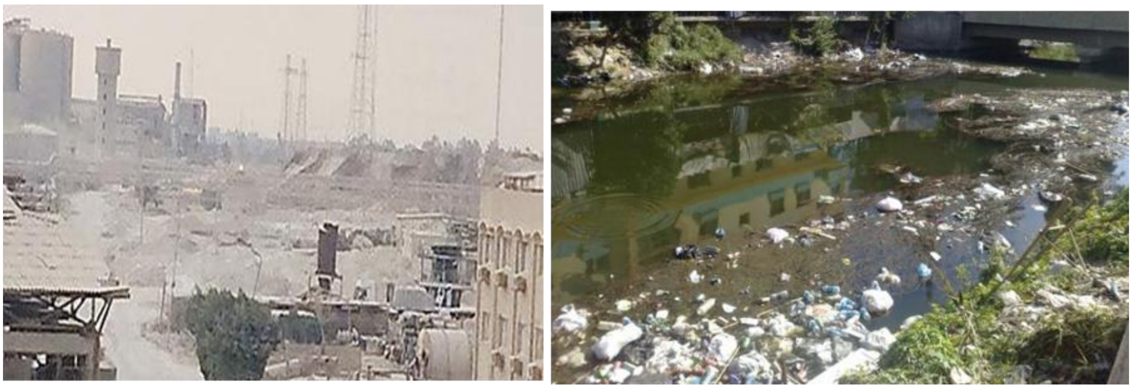

Figure S11. Photos of the contaminant industrial areas.

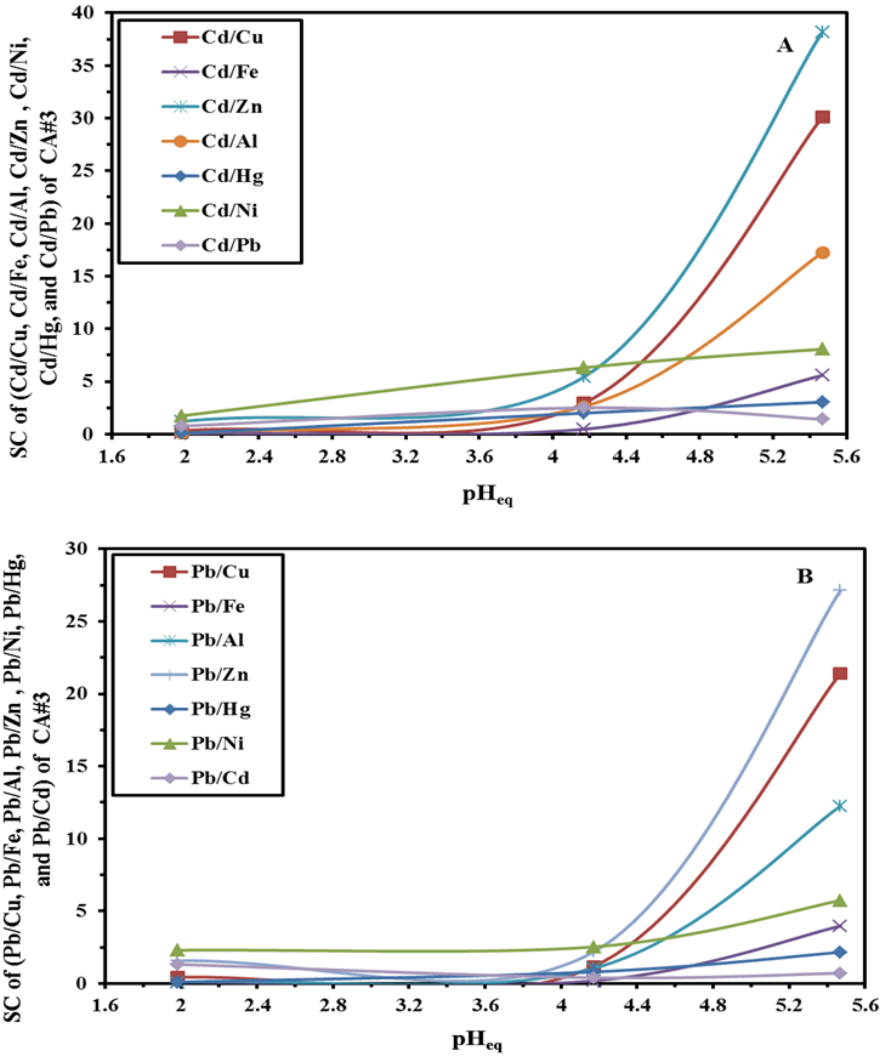

Figure S12. Sorption of Cd(II) (**A**) and Pb(II) (**B**) in naturally contaminated water at different pH values (SD: 1 g L^{-1}; time of agitation: 5 h; speed: 170 (±3) rpm; T: 23 ± 3 °C).

References

1. EU. Council Directive 98/83/EC of 3 November 1998 on the quality of water intented for human consumption. In *Document 31998L0083*; Union, E., Ed. European Union: Brussels, Belgium, 1998.
2. USEPA. *The Drinking Water Standards and Health Advisories*. Office of Water, U.S. Environmental Protection Agency: Washington, DC, USA, 2021; Volume 822-S-12-001, pp. 1–12.
3. WHO. *Guidelines for drinking-water quality*, 4th ed.; World Health Organization: Geneva, Switzerland, 2011; p. 541.
4. Ho, Y.S.; McKay, G. Pseudo-second order model for sorption processes. *Process Biochem.* **1999**, *34*, 451–465, doi:10.1016/S0032-9592(98)00112-5.
5. Tien, C. *Adsorption Calculations and Modeling*; Butterworth-Heinemann: Newton, MA, 1994; pp. 243.
6. Foo, K.Y.; Hameed, B.H. Insights into the modeling of adsorption isotherm systems. *Chem. Eng. J.* **2010**, *156*, 2–10, doi:10.1016/j.cej.2009.09.013.
7. Falyouna, O.; Eljamal, O.; Maamoun, I.; Tahara, A.; Sugihara, Y. Magnetic zeolite synthesis for efficient removal of cesium in a lab-scale continuous treatment system. *J. Colloid Interface Sci.* **2020**, *571*, 66–79, doi:10.1016/j.jcis.2020.03.028.
8. Alexandratos, S.D.; Zhu, X. The effect of hydrogen bonding in enhancing the ionic affinities of immobilized monoprotic phosphate ligands. *Materials* **2017**, *10*, doi:10.3390/ma10080968.
9. Persson, I. Hydrated metal ions in aqueous solution: How regular are their structures? *Pure Appl. Chem.* **2010**, *82*, 1901–1917, doi:10.1351/pac-con-09-10-22.
10. Sato, H.; Yui, M.; Yoshikawa, H. Ionic diffusion coefficients of Cs+, Pb2+, Sm3+, Ni2+, SeO2-4 and TcO− 4 in free water determined from conductivity measurements. *Journal of Nuclear Science and Technology* **1996**, *33*, 950–955.

11. Marcus, Y. *Ion Properties*. Marcel Dekker, Inc.: New York, NY, USA 1997; p. 259.
